# Gelatin methacryloyl advances in regenerative dentistry: A global bibliometric analysis

**DOI:** 10.4317/jced.62741

**Published:** 2025-06-01

**Authors:** Adriana Poli Castilho Dugaich, Andressa da Silva Barboza, Christiane Cabral Leite, Aurélio de Oliveira Rocha, Lucas Menezes dos Anjos, Alexandre Henrique dos Reis-Prado, Marco Cícero Bottino, Juliana Silva Ribeiro de Andrade

**Affiliations:** 1 Department of Dentistry, Federal University of Santa Catarina, Florianópolis (Santa Catarina), Brazil; 2 Department of Cariology, Restorative Sciences and Endodontics, University of Michigan School of Dentistry, Ann Arbor, Michigan, USA; 3 Department of Restorative Dentistry, Universidade Federal de Minas Gerais (UFMG), School of Dentistry, Belo Horizonte, MG, Brazil

## Abstract

**Background:**

This study aims to comprehensively appraise the bibliometric features of articles evaluating the utilization of gelatin methacryloyl (GelMA) in dentistry by conducting a bibliographic search on the Web of Science databases until January 2025.

**Material and Methods:**

The following data were gathered: number and density of citations; authors; year, journal of publication and impact factor; study design and theme; keywords; institution and country of origin. The collaborative network was identified with the VOSviewer software and used to generate collaborative network maps for authors and keywords.

**Results:**

A total of 133 articles reporting the use of GelMA in dentistry were included. The articles were published between 2014 and 2025, and the most prevalent journals were Dental Materials (10,17% and 276 citations) and Acta Biomaterialia (10,1% and 208 citations) The most frequent study designs and themes were laboratory-based in vitro studies (58,6%) and endodontics (37,6%), respectively. Bottino MC (9%; 464 citations) and Dubey N (4,5%; 345 citations) were the most prominent authors in the list. Most articles originated from China (52,6%) and the United States (29,3%). University of Michigan (12,8%) and Sichuan University (6,7%), located in the USA and China, respectively, were the institutions with the most articles. There was a strong negative correlation between the number of citations and the year of publication, with the most recent articles being the most cited.

**Conclusions:**

Global articles related to the use of GelMA in dentistry were published mainly in China, with laboratory-based studies conducted in vitro addressing topics related to the use of GelMA in endodontics.

** Key words:**Gelatin Methacryloyl, Dental Materials, Bibliometric Analysis.

## Introduction

Hydrogels have been an excellent tool for studies in biomedical research, precisely because they have the desirable characteristics for biomaterials, such as biocompatibility, being hydrophilic, being physically and chemically adjustable, in addition to resembling the extracellular matrix (1-7). Notably, hydrogels based on natural polymers outperform their synthetic counterparts, being able to handle their biodegradable components, increasing bioactivity, reducing immune responses, and with superior biocompatibility (1). However, there are still many challenges to be overcome until its effective clinical application, due to its low mechanical resistance and variable degradation rates. Gelatin, a hydrolyzed collagen polymer, can be used as a structural protein in the extracellular matrix of various tissues, but its triple helix structure compromises mechanical strength, which leads to uncontrolled degradation (8).

To deal with the inherent limitations of hydrogel, there is a great tendency to produce chemical cross-linking and functional modifications to enhance the properties of gelatin. GelMA is a gelatin modified through the insertion of methacryl groups (MA), it has gained prominence in studies for having an improved mechanical property and allowing biological functionality (9). Derived from the denatured collagen product, gelatin, GelMA has improved solubility and reduced antigenicity. NoTable features such as Arg-Gly-Asp (RGD) sequences promote cellular interactions and cell remodeling is facilitated by targeting matrix metalloproteinase (MMP)-sensitive sequences, making GelMA a highly attractive biomaterial (10).

In addition to cellular interactions, *in vitro* and *in vivo* tissue morphogenesis is one of the main characteristics of GelMA. It allows to be injected in situ, which demonstrates a minimally invasive technique and in the face of studies, it has shown efficacy in the treatment of periodontal bone defects, regenerative endodontics, dental caries and other applications (7). Because it is very adapTable, GelMA increases the qualities of the scaffolds allowing a biological structure more suiTable for drug administration, tissue repair and cell culture techniques. The crosslinking of GelMA results in hydrogels with remarkable biocompatibility, durability and biomimetic properties, enabling efficient drug administration, as well as improving tissue integration and promoting regeneration. These qualities are especially valuable in applications such as injecTable therapies for the treatment of oral infections, bone regeneration, and regenerative endodontics (4).

The application of bibliometric analysis, a quantitative study design, has immense potential to unveil the scientific scenario of a specific field of knowledge (11,12). This analytical approach consolidates a vast array of studies, extracting crucial data to highlight trends and identify important gaps for further exploration and development in the field assessed (13). Typically encompassing facets such as lead authors, institutions, countries, study designs, themes, and keywords, bibliometric studies not only outline prevailing trends, but also recognize and applaud researchers who actively contribute to the growth of scientific discourse in a given area (14).

Although GelMA hydrogels have been widely used in tissue engineering studies, there is no complete review of their scientific profile in regenerative dentistry. Recognizing the importance of hydrogel and this research gap, the aim of this study was to conduct a bibliometric analysis to explore the scientific landscape, identify emerging trends, and highlight the applications of GelMA in regenerative dentistry. Ultimately, this study is not only intended to reveal the current status of GelMA in regenerative dentistry. It also aims to address the main obstacles and future prospects, thus contributing to the progress of GelMA-based innovations in biomedical fields.

## Material and Methods

The bibliometric review methodology in this study is based on the frameworks of Donthu *et al*. (11) and Dos Anjos *et al*. (12). These works guided the selection criteria and systematic analysis, providing a foundation for the current research. An electronic search was performed on January 13, 2025, in the Web of Science Core Collection (WoS-CC) database (https://www.webofscience.com). The selection of articles was made using the following search strategy: [All=(GelMA OR Gel-MA OR “Methacrylic Gelatin” OR “Gelatin Methacrylate” OR “Gelatin Methacryloyl” OR “Methacrylated Gelatin” OR “Gelatin-methacrylate” OR “Alginate-gelatin Methacrylate”) AND (“Dental” OR “Dentistry” OR “Tooth” OR “Teeth” OR “Dentofacial” OR “Maxillofacial” OR “Orofacial” OR “Dental Materials” OR “Oral Medicine” OR “Dental Science” OR “Oral Research” OR “Oral Diagnosis”)]. Regarding filters, no language or date restrictions were applied. In addition, articles from congresses and studies and articles in which regenerative dentistry was not linked to the main theme were excluded.

For the selection of articles, two independent reviewers (CCL and APCD) were assigned, whose sequential parameters were the revision of the title, the reading of the abstract and the full text, as needed. In cases of controversy, there was a third reviewer (JSR) to reach consensus. For the extraction of bibliometric data, the following parameters were considered: title of the article, year of publication, name of the journal, authors (including all contributing authors and their respective institutions), number of citations, and density of these citations in the WoS-CC. Based on the corresponding author, the institution that generated the article, the country and continent of origin were assigned. The journal’s impact factor for the year 2023 was indicated by Journal Citation Reports. In addition to extracting the keywords, study design and the main theme. Regarding the study designs, a classification was made, in agreement with the reviewers mentioned above, into integrative/narrative reviews, case reports, *in vivo* and *in vitro* laboratory studies. Based on the theme of the study, it was categorized through the most evidenced topics, being classified as: regenerative endodontics, regenerative periodontics, dental materials, implantology, restorative dentistry, oral and maxillofacial microbiology and oral surgery.

The Similarity Viewer (VOSviewer, version 1.6.17.0, Netherlands) was used to identify keywords, authors, and frequency. The other data were identified and added manually, always verified by the independent reviewers (ASB and CCL). The VOSviewer program was used to produce graphic representations and bibliometric networks, evidencing the relationship between the different authors (including only those with at least two occurrences), highlighting the keywords with the most occurrences (considered only with at least three repeated episodes). In the graphic representations, the most frequent keywords were represented with red/orange and more prominent fonts, while green/blue was represented by the keywords with the lowest occurrence. For the authors (3 or more occurrences), bibliometric networks with similar colors were produced to demonstrate collaboration between studies. The authors who published the greater number of studies were represented with the largest circles.

For the network analysis, the clusters were composed of points that were connected to each other and represented by specific colors. The size of the points was directly proportional to the total number of articles related to each co-author, so the largest points, the more relevant the terms were, and the more strongly related to each other, closer they were represented. The thickness of the line connecting the points also changes according to the degree of connection, with the thickest lines representing the strongest connections (15). In the density map, terms with larger focal points, brighter fonts and colors (closer to red) signaled greater occurrences or correlations, while terms with smaller focal points, lighter fonts or colors (closer to yellow or green) suggested fewer occurrences of associated terms.

To evaluate the existence and statistical relevance of the correlation between the number of citations, the impact factor and the year of publication, Spearman’s correlation test was performed, since the Kolmogorov-Smirnov test indicated a non-normal distribution. Statistical analysis was performed by using SPSS software program for Windows (SPSS, version 24.0; IBM Corp).

## Results

- Search results

**Figure 1:**
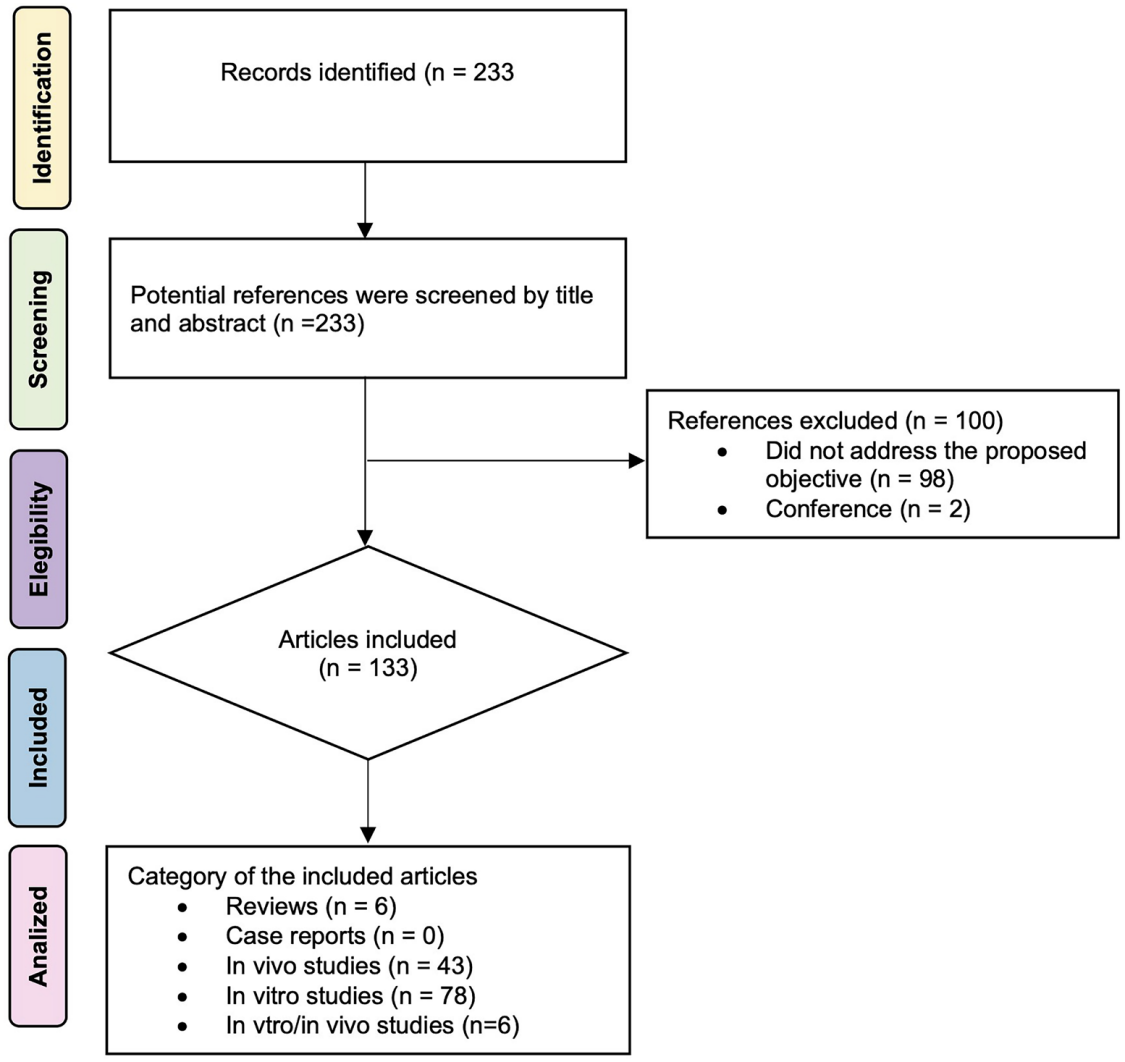
Flowchart for identification and selection of eligible studies for this review. (adapted from PRIMA, 2009).

The search retrieved a total of 233 articles (Fig. 1). All documents related to the use of GelMA in dentistry were examined for consideration of a global bibliometric review. This resulted in 100 articles excluded because they were not related to the proposed theme and two conferences were also excluded. Finally, 133 articles were included. Following this selection process, global articles pertaining to GelMA in dentistry were identified.

- Citation analysis

Global studies were cited 2,786 times in WoS-CC. The citation variation between the studies was from 0 to 155 times. Analyzing the self-citations, 243 times were found, representing 8,7% of self-citations in the WoS-CC. Categorizing in descending order, there were more than seventy citations for the first 10 articles.

The article with the highest number of citations (155 citations) in the WoS-CC, with an *in vitro* study design, was “Photopolymerization of cell-laden gelatin methacryloyl hydrogels using a dental curing light for regenerative dentistry”, by Monteiro *et al*. (2018) (3) in Dental Materials journal, accumulating a mean of 19,4 citations per year. The second most-cited article in the WoS-CC (122 citations) was titled “GelMA-Encapsulated hDPSCs and HUVECs for Dental Pulp Regeneration”, another *in vitro* study, published by Khayat *et al*. (2017) (16), in the Journal of Dental Research, accumulated a mean of 13,5 citations per year. The third most-cited article in the WoS-CC (108 citations) was “Bioprinting-Based PDLSC-ECM Screening for in Vivo Repair of Alveolar Bone Defect Using Cell-Laden, Injectable and Photocrosslinkable Hydrogels”, an *in vivo* study, published by Ma *et al*. (2017) (2), in the ACS Biomaterials Science & Engineering, which obtained an average of 12 citations per year.

- Year of publication

Regarding the year of publication, the first article on the use of GelMA in dentistry to be published was in November 2014, being the oldest found in the search. With the title: “ Enhanced Chondrogenic Differentiation of Dental Pulp Stem Cells Using Nanopatterned PEG-GelMA-HA Hydrogels “, authored by Nemeth and collaborators. The newest was published in 2025, titled “Microenvironment-Regulated Dual-Layer Microneedle Patch for Promoting Periodontal Soft and Hard Tissue Regeneration in Diabetic Periodontitis,” by Qu and collaborators. The years 2023 and 2024 were the ones with the most articles cited (49%) and (n = 67 articles in this period).

- Contributing journals and impact factor

 Table 1 shows the list of journals in which the GelMA articles were published. Ranking in descending order, there were two journals that stand out in first place, Dental Materials with 6 articles published (10,17%) and 276 citations, and Acta Biomaterialia with 6 articles (10,1%) and 208 citations. In the second place was the International Journal of Biological Macromolecules with 5 articles published (8,47%) and 31 citations. Followed by three other journals: Journal of Nanobiotechnology (23 citations), Acs Biomaterials Science & Engineering (180 citations) and Frontiers in Bioengineering and Biotechnology (11 citations) with 4 published articles, corresponding 6,78% each one. Based on JCI reports, the journal with the highest impact factor (IF) in 2023 were: Advanced Functional Materials, with IF 18.5, Matter (IF 18.4) and Bioactive Materials (IF 18).

- Study design and research topics

Most articles were laboratory-based *in vitro* studies (78 articles; 1,470 citations), followed by laboratory-based *in vivo* studies (43 articles; 1,127 citations), *in vivo*/*in vitro* studies (6 articles; 21 citations) and integrative/narrative reviews (6 articles; 65 citations). Most studies addressed the topic “regenerative endodontics” (50 articles; 1,147 citations), followed by “regenerative periodontics” (27 articles; 655 citations), “dental materials” (17 articles; 455 citations), “microbiology” (10 articles; 116 citations), “restorative dentistry” (9 articles; 159 citations), “craniofacial regeneration” (8 articles; 74 citations), “implant dentistry” (7 articles; 55 citations) and “oral surgery” (5 articles; 32 citations).

- Countries and continents

**Figure 2:**
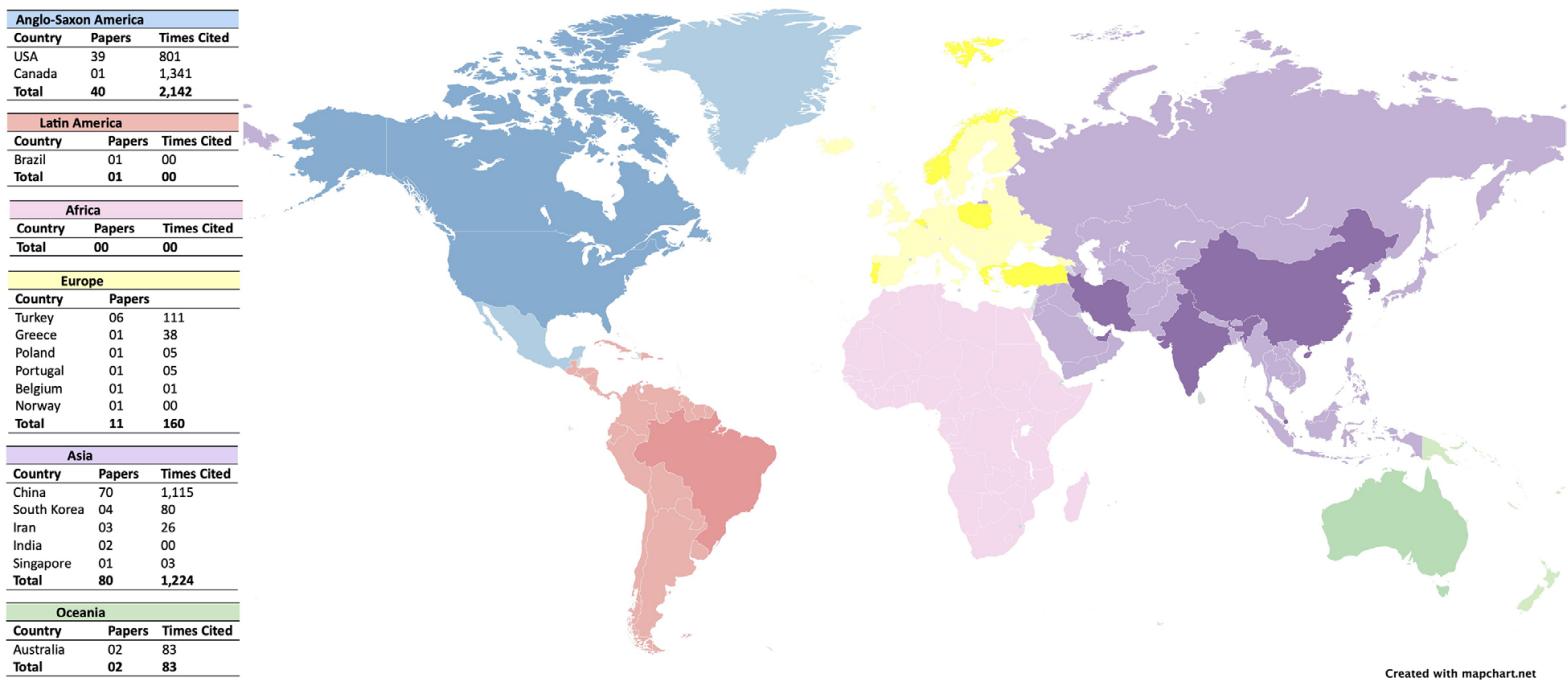
Worldwide distribution of the origin of publications on GelMA in dentistry.

Analyzing the publications in relation to the countries, a total of 15 different countries were compiled. And ranking the number of publications by country, we have: first place China with 70 articles and 1,115 citations, second place the United States of America with 39 articles and 801 citations and third place Turkey with 6 articles and 111 citations. Separating the classification by continents (Fig. 2), we have the Asian continent with the highest number of publications (80 articles; 1,224 citations), North America in second place in number of publications (n=40), but in first place in relation to the number of citations (2,142 citations) according to the WoS-CC and, finally, Europe in third place (11 articles; 160 citations).

- Contribution institutions

 Table 2 presents the top 10 institutions with the highest number of publications. Among the 64 institutions contributing to the top GelMA articles, the top three are the University of Michigan (EUA) with 17 articles and 405 citations, Sichuan University (China) with 9 articles and 239 citations and two institutions were in third place, they are: Shanghai Jiao Tong University (China) with 6 articles and 69 citations, and Zhejiang Chinese Medical University with 6 articles and 64 citations.

- Keywords

**Figure 3:**
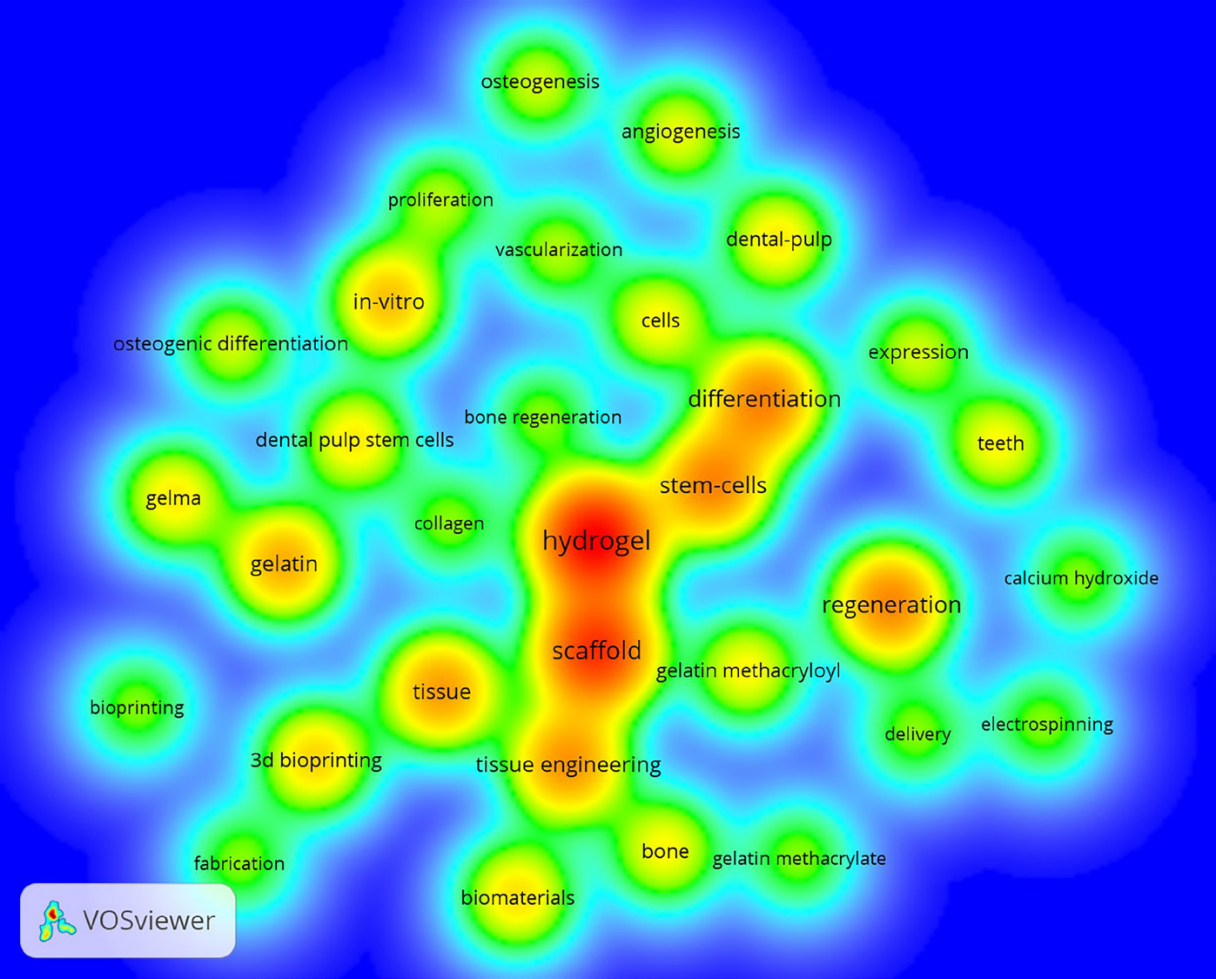
Density map of the main keywords associated with the study. Minimum number of keywords: 3 studies. In the largest points and with highlighted font are the points indicate the relationship and use of these words in the same studies.

A total of 558 keywords were found. The most pronounced word was “Hydrogel” (n=46), in second place was “Scaffold” (n=37), followed by “Stem cells” (n=23) and “Differentiation” (n=23). Figure 3 shows the co-occurrence of the words used by the authors. Keywords with 5 or more occurrences have been inserted into the image.

- Contributing authors

**Figure 4:**
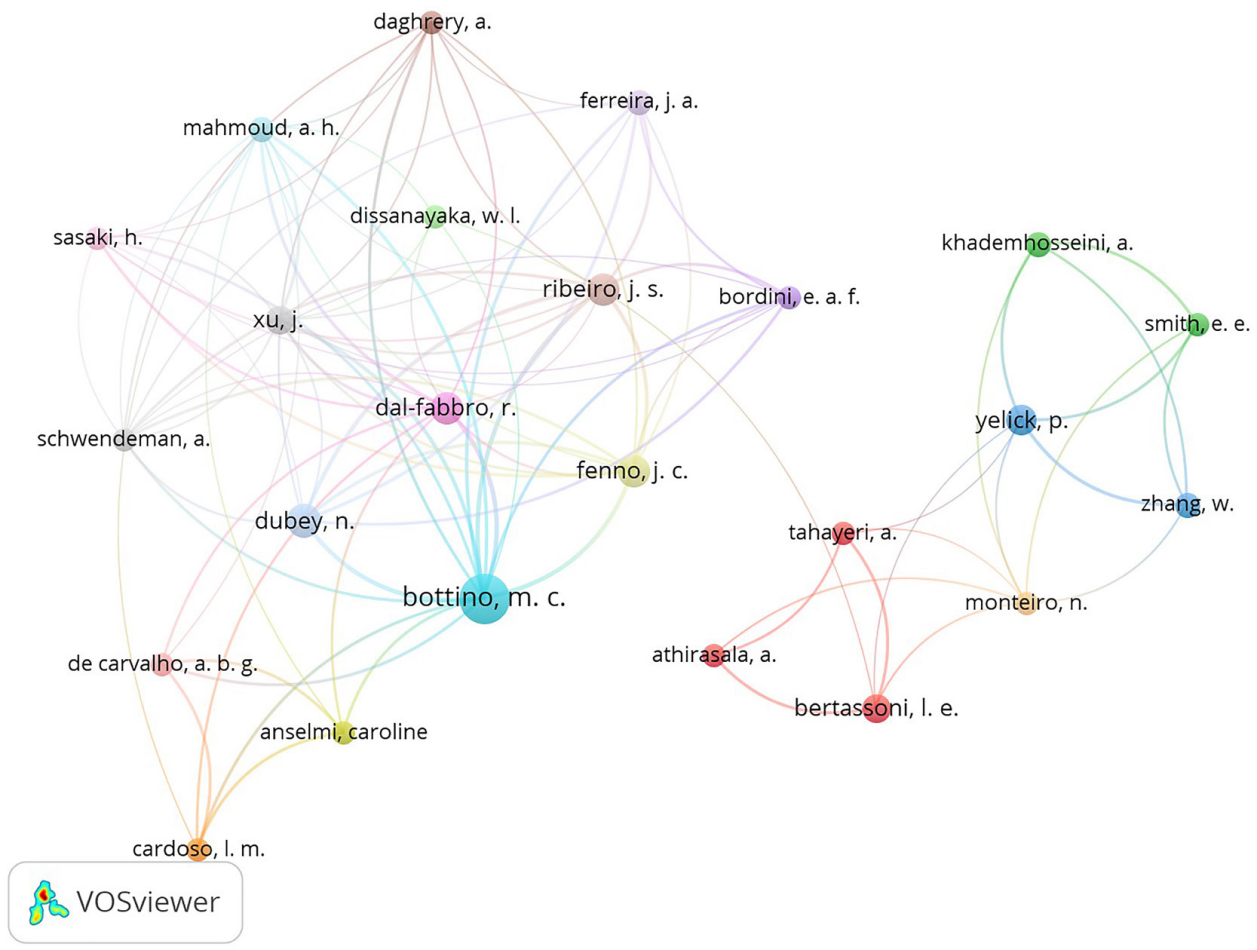
Density map of authors and collaborative co-authorship among them.

A total of 200 authors were found with publications related to GelMA.  Table 3 presents the ranking of the top 10 authors with the highest number of publications. Bottino MC (18 articles; 464 citations) was the most cited author, followed by Dubey N (9 articles; 345 citations), Ribeiro JS (8 articles; 267 citations), and Fenno JC (8 articles; 218 citations). Figure 4 shows the frequency and relationship of co-authorship among the main authors.

## Discussion

Aiming at the construction and manipulation of cellular frameworks, hydrogels are considered an alternative capable of meeting the demands for specific applications. These hydrogels are constructed, primarily for biomedical purposes in regenerative medicine, for drug delivery, and even as tissue adhesives. And they can come from natural or synthetic polymers with various types of crosslinking. GelMA can be produced to mimic biological tissue, making it very interesting for tissue engineering and biological regeneration (7).

Due to this, numerous scientific studies can be observed in the literature investigating its use in dentistry (5,6). However, no bibliometric review analyzing this topic was observed in the literature. Therefore, the objective of the current bibliometric study was to select and evaluate the scientific profile of the most cited articles that address the use of GelMA in regenerative dentistry. The most cited articles were mainly of the laboratory type, developed mostly at the University of Michigan (USA), normally addressing as an endodontic topic.

It is noteworthy here that the journal’s impact factor was not related to the number of citations, demonstrating that an article would not necessarily be cited more simply due to the relevance of the journal in which it was published. Furthermore, the statistical analysis of citation density demonstrated that the most recent articles received the highest number of citations, indicating a growing interest and impact of GelMA research in regenerative dentistry. One hypothesis for this result would be due to the topicality of the subject where innovations are becoming more constant every day.

The number of citations reflects a study’s importance and is commonly used to evaluate its scientific impact (7). In some research fields, an article with over 100 citations is often considered a classic (18). Focusing on the recent application of GelMA in regenerative dentistry, it was found that, among the 133 articles analyzed, only three of them had more than 100 citations, though the fourth-ranked article was approaching this threshold in WoS-CC. Compared to other bibliometric studies, this research revealed a relatively low rate of self-citations. Self-citation has been described as “a marker of productive authors,” with the likelihood of self-citation increasing as an author publishes more articles (13,19,20). However, unethical behaviors, such as excessive and unnecessary self-citation, are criticized for artificially boosting metrics through self-promotion (21).

The article with the highest number of citations (155 citations) in the WoS-CC, with an *in vitro* study design, was “Photopolymerization of cell-laden gelatin methacryloyl hydrogels using a dental curing light for regenerative dentistry”, by Monteiro *et al*. (2018) (3) in Dental Materials journal. This article showed that the combination of dental cells with hydrogels photopolymerized with visible light is a promising strategy for regenerative endodontics, facts that justify the highlight in the number of citations in this article. However, coincidentally, this study also led with the highest density of citations, which states that its merit in citations may be linked to the year of publication, as being a very current subject, the most recent articles are the most cited. As a result, the highest number of citations was observed in the years 2023 and 2024.

Founded in 1985, Dental Materials (Dent Mater) is published on behalf of the Academy of Dental Materials. This scientific journal, produced by Elsevier, serves as the official publication of the Academy. Its primary goal is to promote the swift exchange of scientific knowledge between academia, industry, and dental professionals. It is the journal that has published the most studies related to GelMA along with Acta Biomaterialia (Acta Biomater), which aims to cover the relationship between the structure and function of the biomaterial at all length scales. The importance of these journals for the clinical and scientific community justifies their intense occurrence in this and other bibliometric analyses (13,22).

It is desirable that the studies begin to be developed in the laboratory, as they allow for a precise project with the possibility of controlling and operationalizing most of the variables. In addition to enabling varied measurements and manipulations, resulting in more accurate response rates (18). Hydrogels are biodegradable and have malleability, low toxicity and ease of modifying their structure, allowing combinations with biomolecules that have regenerative properties, making these materials desirable for bone tissue engineering (4,6). Which explains the relevance of studying this material. However, only six reviews were identified. Reviews can exert a significant influence among researchers and professionals, since from a careful analysis of reviews it is possible to modify clinical practice or even modify lines of research in centers of excellence (23). One hypothesis to justify the small number of revisions would be the topicality of the subject. It can be noted that of all the articles cited, most are from 2020 to 2024. In this sense, there is a great opportunity and demand for more studies of GelMA in dentistry.

The area with the most studies on GelMA in dentistry was endodontics, with a significant number of investigations focused on pulp regeneration. Most research has aimed to identify a reliable and clinically applicable method for revascularization and pulp regeneration (16). Periodontics ranked second, followed by dental materials. Given the complex and diverse nature of oral health problems, there seems to be an ongoing need for dental biomaterials that can interact effectively across different tissues and anatomical regions. This justifies the interest in developing a hydrogel capable of helping in the regeneration of oral tissues (2). Similar to other hydrogels documented in the literature, regarding use as a drug carrier, GelMA can be combined with several additives, including nanotubes and biomolecules (4). Despite these encouraging results, there are some limitations to the use of the hydrogel, which include finding a sturdy enough and biodegradable scaffold at the optimal time that effectively replaces the extracellular matrix of the natural pulp, in addition to allowing adequate blood supply in a timely manner to ensure the survival of transplanted cells *in vivo* or that allows efficient recruitment of host cells for tooth revitalization (16). The fact that GelMA proves to be a candidate for the use of this drug delivery and cell revitalization system justifies highlighting the most cited research evaluating this substance as a solution for tissue regeneration in dentistry.

According to the results of this literature review, the USA was ranked first in number of citations (801), while China ranked first in terms of the number of published articles related to GelMA (70). This is likely due to the fact that both the U.S. and China are home to major research and innovation centers, investing heavily in new technologies, and boasting prestigious universities with high volumes of annual publications.

The most frequently cited author was Bottino MC, who proposed that integrating a highly porous polycaprolactone (PCL) fibrous mesh with a well-defined 3D architecture into a hydrogel containing additively manufactured (AMP) components can regulate mechanical properties and improve the osteogenic potential of GelMA. This offers a promising avenue for GelMA to be used as a bioactive membrane for bone regeneration. Following Bottino, Dubey N explored the drug release kinetics from hydrogels loaded with metformin-infused mesoporous silica nanospheres. Ribeiro JS made a noTable contribution as the first to report the synthesis and clinical potential of a nanotube-modified, injectable, biodegradable, and biocompatible on-demand (MMP-responsive) GelMA hydrogel system for intracanal CHX administration, offering a viable strategy for dental infection ablation. Lastly, Fenno JC successfully developed an injecTable and photocrosslinkable GelMA hydrogel with short ciprofloxacin-eluting nanofibers (CIP) for the treatment of oral infections.

The most prevalente keyword was “hydrogel” followed by the word “scaffolds” demonstrating here a new strategy to design a hydrogel scaffold loaded with cells or drugs to act a target tissue. Thus, using the moldable physical and mechanical properties of GelMA to analyze internal conditions (microstructure, modulus of elasticity and damping) to increase the viability and cell proliferation of cells that mimic odontoblasts, endothelial cells and drugs. One possible reason why the term “GelMA” is not the most cited in this review may be the fact that GelMA is a specific hydrogel, and different types can be found in the literature. The term GelMA is not standardized in literature, so several terms can be used to name the same product (hydrogel, gelatin, methacryl gelatin), which was evidenced in this study, which showed the predominance of these terms in the compilation of results, as shown in Figure 4.

The thorough examination of the key characteristics of the articles represents a noteworthy strength within this study. The deliberate choice to forgo year or language filters in the literature search allowed for a more expansive and inclusive exploration. The exclusive utilization of WoS-CC (Web of Science Core Collection) in this study is based on its acknowledged reputation for comprehensive coverage and prestige in bibliometric analyses, aligning with established practices in dentistry demonstrated by prior studies (23-25). This strategic decision ensures methodological consistency, enabling meaningful comparisons and enhancing the overall reliability of the study by adhering to established norms in the field. By concentrating on a single, widely recognized database, the study aims for uniformity and coherence in analyzing trends in dental research, a successful approach observed in other repuTable works. Citing studies that use a similar approach highlights the reliability and significance of WoS-CC in identifying key publications and trends in dental research, greatly enhancing the overall credibility of the study. However, it is important to recognize a potential limitation of this research: the exclusive dependence on WoS-CC as the primary search platform. While this decision is made with careful consideration and might be perceived as a constraint, it aligns with precedents set by significant bibliometric analyses within the field of dentistry (19-25). The rationale behind this selection is grounded in the widely recognized prestige of WoS-CC as the go-to database for conducting rigorous and reliable bibliometric studies, as emphasized in previous scholarly works (23-25). In essence, this strategic approach enhances the study’s credibility and ensures a robust analysis of dental research trends.

## Conclusions

This bibliometric review aimed to elucidate the recent state and research trends pertaining to the utilization of GelMA in the field of dentistry. From what we know based on the results presented, at the moment it is possible to see a greater research interest in North America, although China is the country highlighted with the largest number of articles on the topic. Most of the works were published in Dental Materials and Acta Biomaterialia, with the majority being *in vitro* laboratory-based research projects, mainly investigating the use of GelMA in pulp regeneration. A limited number of reviews on the use of GelMA in endodontics were identified, highlighting the potential significance of this topic for future research endeavors. Based on the data collected, it is possible to have a broad vision in the direction of potential studies and in the planning of future applications for GelMA in dentistry.

## Figures and Tables

**Table 1:** Journals that published the most on GelMA in dentistry.

Source title	Number of papers	Impact factor
ACTA BIOMATERIALIA	6	9.4
DENTAL MATERIALS	6	4.6
INTERNATIONAL JOURNAL OF BIOLOGICAL MACROMOLECULES	5	7.7
JOURNAL OF NANOBIOTECHNOLOGY	4	10.6
ACS BIOMATERIALS SCIENCE & ENGINEERING	4	5.5
FRONTIERS IN BIOENGINEERING AND BIOTECHNOLOGY	4	4.3
ADVANCED HEALTHCARE MATERIALS	3	10.0
MATERIALS TODAY BIO	3	8.7
ACS APPLIED MATERIALS & INTERFACES	3	8.5
JOURNAL INTERNATIONAL OF BIOPRINTING	3	6.8
INTERNATIONAL JOURNAL OF NANOMEDICINE	3	6.7
JOURNAL OF MATERIALS CHEMISTRY B	3	6.1
JOURNAL OF DENTAL RESEARCH	3	5.7
INTERNATIONAL JOURNAL OF MOLECULAR SCIENCES	3	4.9
POLYMERS	3	4.7
JOURNAL OF BIOMEDICAL MATERIALS RESEARCH PART A	3	3.9

**Table 2:** The top 10 institutions with the highest number of publications.

Institution	Publication
University of Michigan	17
Sichuan University	9
Shanghai Jiao Tong University	6
Zhejiang University	6
Tufts University	5
Oregon Health & Science University	4
University of Hong Kong	4
Wenzhou Medical University	4
China Medical University	3
Harvard University	3
Jilin University	3
Middle East Technical University	3
Peking University	3
Xi'an Jiaotong University	3

**Table 3:** Authors with more publications on GelMA in dentistry.

Authors	Number of articles	Number of citations
Bottino MC	18	464
Dubey N	9	345
Ribeiro JS	8	267
Fenno JC	8	218
Dal-Fabbro R	8	83
Tian W	8	228
Yelick P	7	269
Xu J	6	129
Bertassoni LE	6	328
Mahmoud AH	5	76

## Data Availability

The datasets used and/or analyzed during the current study are available from the corresponding author.
